# Department of Canine, Feline, and Avian Medicine and Surgery

**Published:** 1901-08

**Authors:** Cecil French

**Affiliations:** Washington, D. C.


### DEPARTMENT OF CANINE, FELINE, AND AVIAN MEDICINE AND SURGERY.

By Cecil French, D.V.S.,
WASHINGTON, D. C.

Puerperal Septic Metritis. Perforation of Uterine Wall and Purulent Peritonitis. Cœlio-oöphorohysterectomy. Recovery. A toy black-and-tan terrier, weighing four pounds heavy, aged two years, was delivered to me one Monday morning for treatment, accompanied with the following history: On the preceding Wednesday a sanguineous vaginal discharge had appeared and had continued until the Saturday, when a fully-developed but decomposing fœtus was born without much difficulty. Full term of nine weeks would have been reached the day I received the animal. On Sunday a second decomposed fœtus was delivered with considerable difficulty, the owner assisting. Early on Monday morning the animal showed signs of impending collapse, being hardly able to keep to its feet and occasionally vomiting.

When it arrived at my office I found there was a discharge of foul, purulent matter taking place from the vagina. Temperature was subnormal, pulse 160, and one spell of vomiting occurred, the vomitus consisting of semi-feculent matter. The abdomen was somewhat painful to pressure.

Here was an animal in a condition which, to terminate favorably, demanded prompt surgical treatment. The discharge was undoubted evidence of putrefactive changes going on within the uterus. Of course, it was impossible to know to what extent such changes had proceeded, but the systemic collapse pointed to absorption of septic products, which, if not cut short, and that quickly, could lead to no other termination than death. Puerperal septic metritis is a disease which runs a rapid course and invariably terminates fatally. The inflammatory process extends from the mucosa, and soon involves all the coats of the organ, and local ulcerative degenerations occur and lead to perforation.

I consider the treatment of antiseptic irrigation advocated by Müller to be utterly useless, experience having taught me that nothing short of total removal of the infecting centre is of any avail.

I administered morphine, one-eighth grain, hypodermatically, and one hour later placed the animal on the operating-table. Complete anæsthesia was induced with a few inhalations of chloroform, and I opened the peritoneal cavity in the median line in the usual manner. There immediately gushed forth a considerable quantity of sanious and slightly purulent liquid. On raising and bringing both cornua out of the wound the pressure of my fingers caused an exudation of thick fetid pus through two of several necrotic foci or ulcers which could be seen dotted here and there over the organ. These two foci had evidently become permeable a few hours previously, and accounted for the slight purulence of the fluid contained in the peritoneal cavity. I placed a stout silk ligature on the proximal side of the ovaries, another around the anterior extremity of the vagina, and excised the intervening tissues, which comprised the cervix, body, and both cornua of the uterus, Fallopian tubes, and ovaries. Ordinarily it is best to ligate both utero-ovarian arteries separately, but in very small animals this is hardly necessary, so I dispensed with technique which might unnecessarily prolong the operation. The ovaries should always be removed whenever the uterus is extirpated, for reasons which I pointed out in the May number of this JOURNAL. I next turned my attention to irrigating the peritoneal cavity, which by this time was greatly contaminated with purulent matter which had exuded from the diseased organ. This was accomplished with the aid of a reservoir syringe. For two or three minutes a stream of moderately hot water (which, however, had not been sterilized) was played over all the visceral organs in the neighborhood of the extirpated parts, and then the cavity swabbed out with absorbent cotton. Having got everything sweet and clean, I could ascertain what degree of inflammation the peritoneum had suffered. This was not very apparent, though here and there were patches of redness. I then sewed up the muscular wall, using continuous suture of silver wire. At this stage the animal showed marked symptoms of collapse, not from chloroform, for I had withdrawn that immediately after unconsciousness had been established, but from shock. I injected trinitrin, one one-hundredth of a grain, hypodermatically, and hastily procuring a handful of salt dissolved the latter in some hot water. This was a rough guess at a normal salt solution. I then inserted the blunt needle of an aspirating syringe through the stitched wound and well within the peritoneal cavity, and injected this solution every two or three minutes as fast as it was absorbed. In all one and one-half ounces were injected. Within ten minutes the effect was marvellous, the pulse slowing up and increasing in volume, and the little creature making strenuous efforts to release herself from restraint.

Fearing infection of the subcutis, consequent on contamination with the matter that had passed by it, I did not close the skin wound, but packed it with gauze and xeroform.

I covered the animal up with a blanket, and in half an hour she was warm and resting easily.

The evening of the following day she partook of nourishment, and subsequently made an uneventful recovery.

Two points are emphasized by the success of this case. It demonstrates, firstly, the wisdom of making an early explorative celiotomy, with removal of the diseased parts, if necessary ; and, secondly, the remarkable recuperative power of salt solution injected intraperitoneally.

The Dentition of Two Mexican Hairless Dogs. In the November, 1900, number of this Journal I recorded an observation of the structure and arrangement of the teeth of a member of the breed in question, and also cited other authorities. I have recently had an opportunity to examine the jaws of two more members of this breed—a male and a female. The male showed better development than the female. He was possessed of the following teeth (aged, one year) : In the *upper* jaw there were four fairly well-developed incisors, but without the cuspid arrangement. Two well-developed canines were in position, and on either side well-developed first premolars and first molars. There were no other teeth in this (upper) jaw, hence the animal was minus the superior sectorial, which is to be regarded as the most important tooth in the head, the animal being solely dependent on it for crushing and dividing tough animal membranes.

In the *lower* jaw I found four incisors developed in about the same degree as those in the upper. There was only one canine present, and that rudimentary and on the left side. Closely hugging it posteriorly was evidently an unshed deciduous tooth, which is commonly to be observed in young dogs. First and second premolars and first and second molars were in evidence, so that the lower jaw possessed its sectorial. All the molars showed better development than the incisors. In the female (aged, two years) there was no trace whatever of either incisors or canines, though there was a kind of continuous indentation or slit in the gum where the teeth should have been in place.

In the *upper* jaw there were first and third premolars on either side. Here, again, was absence of upper sectorials.

In the *lower* jaw I found only first and second molars on either side, showing inferior sectorials intact, as in the male.

As was natural to expect, I learned from the owner that neither animal cared to eat hard foods, but preferred slops and soft bread and cakes. Meat could only be swallowed if cut fine, as the animals were without the ability to reduce the size of tough or hard articles of diet, which dogs with normal mouths accomplish by the scissor-like movements of their sectorials.

---

Systemic Secretion of the Testicle. Brown-Séquard demonstrated that the testicle elaborates two distinct secretions: the semen which is *excrementitial* and another *recrementitial.* The latter, whose identity is not determined, is absorbed by the blood and acts as a special excitant upon which virility or the activity of the procreative functions depend. In order to determine if after suppressing one of these secretions the other would continue the vas deferens was ligated; the testicle continued to develop and secrete spermatozoa.

Pruneau, in order to determine the effects of the resorption of the recrementitial secretion upon the organism, operated upon immature dogs, rabbits, and guinea-pigs in which spermatogenesis had already commenced as well as upon adult animals. He concludes: Section of the vas deferentia slightly diminishes the urinary secretion, but is without influence upon the prostates in the normal animal. There is a marked atrophy of the seminal vesicles. The total destruction of nitrogen and the elimination of urea were notably diminished. As to the blood, the results were contradictory: in young animals its weight was slightly increased; in the old, diminished. In both groups the weight of the skeleton was distinctly increased. The functions of relation were more active; the animals operated on were more vigorous. Section and ligation of the vas deferens, in consequence of the resorption of the semen, therefore seems to produce results analogous to those obtained from the injection of testicular liquid, but its effects are slow and continuous.—*Annales de Méd. Vét.*

